# Carbon-Based Electrocatalysts Derived From Biomass for Oxygen Reduction Reaction: A Minireview

**DOI:** 10.3389/fchem.2020.00116

**Published:** 2020-02-28

**Authors:** Mi Wang, Shiyu Wang, Haoqi Yang, Wen Ku, Shuchen Yang, Zhenning Liu, Guolong Lu

**Affiliations:** ^1^Engineering College, Changchun Normal University, Changchun, China; ^2^Key Laboratory of Bionic Engineering (Ministry of Education), College of Biological and Agricultural Engineering, Jilin University, Changchun, China

**Keywords:** oxygen reduction reaction, biomass-derived, electrocatalyst, fuel cells, hetero-catalysis

## Abstract

Oxygen reduction reaction (ORR) electrocatalysts derived from biomass have become one of the research focuses in hetero-catalysis due to their low cost, high performance, and reproducibility properties. Related researches are of great significance for the development of next-generation fuel cells and metal-air batteries. Herein, the preparation methods of various biomass-derived catalysts and their performance in alkaline, neutral, and acidic media are summarized. This review clarifies the research progress of biomass carbon-based electrocatalysts for ORR in acidic, alkaline and neutral media, and discusses the future development trends. This minireview can give us an important enlightenment to practical application in the future.

## Introduction

The development of green energy has been urgent due to the increasingly international attention toward energy shortages and environmental pollutions. Among these energy storage devices, fuel cells have been considered as a promising alternative with clean, stable, and sustainable properties in order to meet the growing global energy demands (Dai et al., 2015). Therefore, rational design of low-cost oxygen reduction electrocatalysts is critical for the storage and electrochemical performance of renewable energy sources (Liu et al., 2015). As an ideal component of primary energy equipment, fuel cells using hydrogen or hydrocarbon fuels can directly convert chemical fuel into electricity through electrochemical processes and operate at ambient temperature (Winter and Brodd, 2004). Even though the amount of Pt is capable of achieving the desired catalytic effect by using Pt alloys (Stamenkovic et al., 2007; Jiang et al., 2009) or making core-shell nanostructures with supporting materials (Li et al., 2018), the high cost, insufficient durability, and unrefined technology still restrict the practical large-scale commercialization (Guo et al., 2013; Kaur et al., 2019).

**Figure 1:**
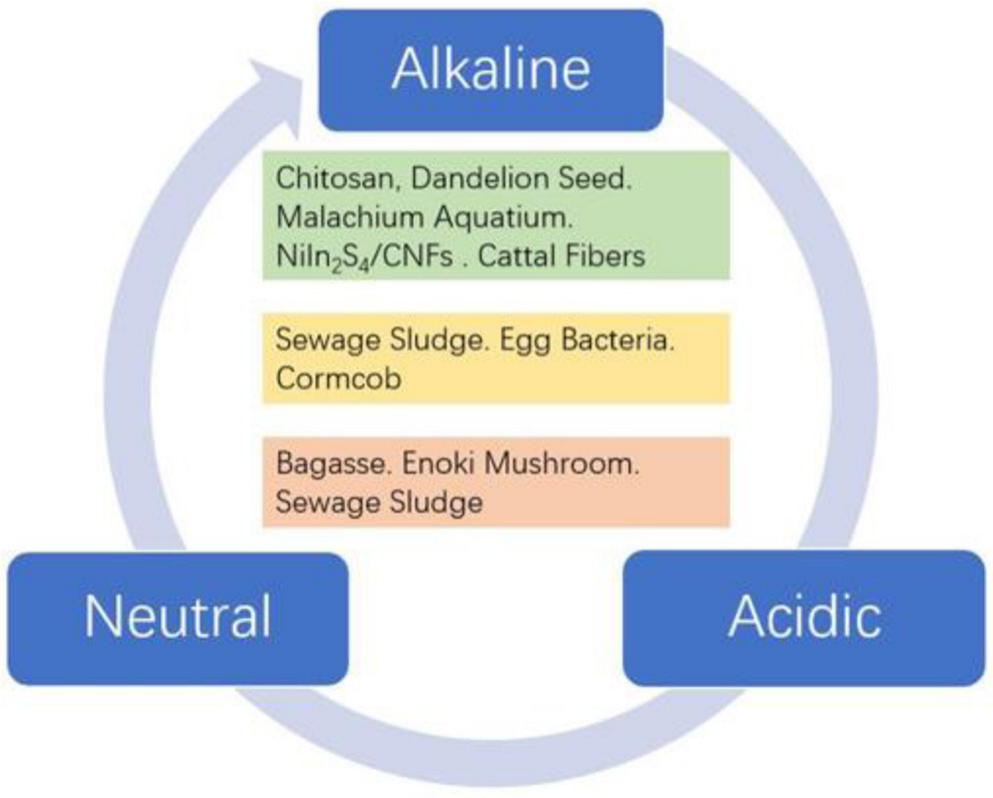
Overview of various biomass precursors in different media.

Accordingly, to address these above issues, numerous non-Pt materials have been studied as cathode catalysts alternative to Pt-based catalysts for ORR (Banham et al., 2015; Shao et al., 2016). Cuurently, biomass-derived materials, such as active carbon (Deng et al., 2010), enzyme (Qiao et al., 2010), microorganism (Majidi et al., 2019; Papiya et al., 2019), transition metal porphyrins (Zheng et al., 2016), NiIn_2_S_4_/CNFs (Fu et al., 2019), and phthalocyanines (Kaare et al., 2016; Bhowmick et al., 2019) have potential capability to replace Pt. Therefore, the method of producing ORR catalyst from biomass has attracted extensive attention of researchers in many aspects (Liu et al., 2016; Sawant et al., 2016; Zhao et al., 2017). In this minireview, the catalytic mechanism for oxygen reduction reaction in different media is given, including a four-electron pathway and a two-electron pathway. Thereafter, various biomass-derived carbons and composites for ORR in alkline, neutral, and acidic media are summarized (Figure 1). The aforementioned biomass-derived carbons and composites exhibit outstanding electrochemical performance, which make them promising candidates for alternating Pt-based electrocatalysts. Moreover, we discuss the furture research direction and challenges of biomass-derived carbon electrocatalyst for ORR in different media.

## Catalytic Mechanism for Oxygen Reduction Reaction

Generally, the oxygen reduction reaction in an aqueous electrolyte can proceed via two types: a four-electron pathway and a two-electron pathway. The former method could directly reduce oxygen to water, which is preferable than the two-electron route using hydrogen peroxide as a reaction intermediate. The choice of overall pathway depends on the type of catalyst. So far, many literatures have reported the use of biomass and its derivatives as ORR catalysts in neutral or alkaline medium, the reaction mechanism can be described as follows:

Four-electron pathway:

$${\text{O}}_{2}+{\text{2H}}_{2}\text{O}+{\text{4e}}^{-}\to {\text{4OH}}^{-}\text{ E}=\text{0}.401\text{ V}$$

Two-electron pathway:

$$\begin{array}{l} {\text{O}}_{2}+{\text{H}}_{2}\text{O}+{\text{2e}}^{-}\;\to \;{\text{HO}}_{2}^{-}\;+\;{\text{OH}}^{-}\text{ E}=-\text{0}.065\text{ V }\\ {\text{HO}}_{2}^{-}+{\text{H}}_{2}\text{O}+{\text{2e}}^{-}\to {\text{3OH}}^{-}\text{ E}=\text{0}.867\text{ V}\\ {\text{2HO}}_{2}^{-}\;\to \;{\text{2OH}}^{-}+{\text{O}}_{2} \end{array}$$

The accumulation of OH^−^ at the catalytic sites can lead to a considerable decline in the kinetic performance (Popat et al., 2012).

In an acidic medium, the mechanism can be described as follows:

Four-electron pathway:

$${\text{O}}_{2}+{\text{4H}}^{+}+{\text{4e}}^{-}\to {\text{2H}}_{2}\text{O E}=\text{1}.229\text{ V}$$

Two-electron pathway:

$$\begin{array}{l} {\text{O}}_{2}+{\text{2H}}^{+}+{\text{2e}}^{-}\to {\text{H}}_{2}{\text{O}}_{2}\text{ E}=\text{0}.695\text{ V}\\ {\text{H}}_{2}{\text{O}}_{2}+{\text{2H}}^{+}+{\text{2e}}^{-}\to {\text{2H}}_{2}\text{O E}=\text{1}.770\text{ V}\\ {\text{2H}}_{2}{\text{O}}_{2}\to {\text{2H}}_{2}\text{O}+{\text{O}}_{2} \end{array}$$

## In Alkaline Medium

Catalysts with excellent ORR performance in an alkaline medium will play an important role in metal-air batteries. Currently, biomass-derived carbon electrocatalysts for ORR have been reported to be the most effective in alkaline medium. As illustrated in Figure S1A, natural cattail fibers are used to prepare porous nitrogen-doped carbon through direct chemical activation and heteroatom doping (Liu et al., 2019). The obtained graphene-like sheets from biomass pyrolysis are assembled into three-dimensional carbon frameworks, which exhibit a significant synergistic effect on the improvement of catalytic properties. Fu et al. successfully prepared NiIn_2_S_4_ nanosheets supported on carbon nanofibers (Figure S1B). It was found that the performance of prepared catalyst is better than that of single metal Ni or In sulfides (Fu et al., 2019). Corn stover was also reported as a biomass precursor for the preparation of nitrogen, cobalt co-doped electrocatalyst (NCAC-Co) for ORR and aluminum-air batteries (Liu et al., 2018). The resulting porous biocarbon electrocatalyst not only exhibits the 4-electron oxygen reduction mechanism, but also displays excellent durability and stability. The author clearly demonstrated that the NCAC-Co has good prospects and is expected to become an economical and large-scale catalyst substitute for metal-air batteries. As illustrated in Figure S1C, the NCAC-Co electrocatalyst was prepared by two major steps of KOH activation and heteroatom doping. In addition to the examples metioned above, other promising biomass precursors are dandelion seeds (Tang et al., 2019), shaddock peel (Lu et al., 2019), chitosan (Zhao et al., 2017), mulberry leaves (He et al., 2019), gelatin (Yang et al., 2019), and chitin (Wang et al., 2019) etc.

## In Neutral Medium

As the research on microbial fuel cells becomes more and more in-depth, the development of new high-performance biomass carbon materials has become increasingly crucial. Compared with the alkaline medium, biomass catalysts have fewer applications in neutral media because the ORR performances were slightly negative. However, biomass-derived electrocatalysts have a higher stability in a neutral medium. For instance, the sewage sludge-derived biochar was successfully prepared and employed as an excellent ORR electrocatalyst. As shown in Figure S2A, the structural change of as-obtained carbonized materials was clearly observed, which was detected by Raman spectroscopy (Yuan et al., 2013). In addition, Lu et al. developed a low-cost method to prepare egg-based heteroatom-doped carbon catalysts. The ORR catalytic activity of prepared electrocatalyst in a neutral medium is comparable to that of a commercially available Pt/C catalyst (Figure S2B, Lu et al., 2017). In the neutral medium, not only the above-mentioned biomass precursors are successfully used, the corn cob-derived catalysts synthesized by a simple pyrolysis method (Li et al., 2018) and mesoporous Fe-NC electrocatalysts prepared by activation of bacteria growing on Fe minerals (Ma et al., 2017) also delivered superior electrochemical performance.

## In Acidic Medium

Owing to its low cost and abundant sources, biomass carbon materials can be used as excellent cathode catalysts to replace noble-metal electrocatalysts. One typical material is nitrogen-doped nanoporous carbon flakes extracted from low-cost bagasse (Yuan et al., 2016). Moreover, the enoki mushroom derived carbon electrocatalyst also possesses outstanding ORR activity and durability. The significant difference in ORR activity of the two carbon materials is shown in Figure S3A. Extraction of N-doped carbon nanomaterial from Nenoki mushroom biomass in a certain temperature is shown in Figure S3B (Guo et al., 2015). In addition to the above examples in an acid medium, sludge-based multi-doped electrocatalysts (Yuan and Dai, 2016) and corn starch derived nitrogen-doped carbon electrocatalysts (Wang et al., 2012) are also utilized for ORR in acidic medium.

As we discussed above, the biomass-derived materials not only have outstanding contributions in terms of catalytic performance, but also promote environmental improvement. Therefore, we establish a link between the electrolytes, biomass precursors, optimal preparation temperature, specific surface area, and onset potential of various ORR electrocatalysts, which are listed in Table 1.

**Table 1:** Typical examples of electrocatalysts derived from biomass.

**Electrolyte**	**Biomass precursor**	**Optimal preparation temperature**	**Specific surface area**	**Onset Potential of Catalyst/Pt/C**
0.1 M KOH	Chitosan (Zhao et al., 2017)	800°C	543 m^2^ g^−1^	−0.08/−0.055 V (vs. Ag/AgCl)
0.1 M KOH	Corn Stovers (Liu et al., 2018)	900°C	1877.3 m^2^ g^−1^	/
0.1 M KOH	Dandelion Seed (Tang et al., 2019)	900°C	1324.1 m^2^ g^−1^	0.83/0.85 V (vs. RHE)
0.1 M KOH	Chrysanthemum Flowers (Xu et al., 2017)	800°C	810 m^2^ g^−1^	1.0/1.0 V (vs. RHE)
0.1 M KOH	Malachium Aquaticum (Huang et al., 2016)	900°C	851.41 m^2^ g^−1^	−0.053/−0.043 V (vs. Ag/AgCl)
0.1 M KOH	Microalgae (Wu et al., 2019)	1,000°C	/	1.0/1.0 V (vs. RHE)
0.1 M KOH	Lotus Root (Rajendiran et al., 2019)	700°C	884 m^2^ g^−1^	0.84/0.92 V (vs. RHE)
0.1 M KOH	Shaddock Peel (Lu et al., 2019)	900°C	548 m^2^ g^−1^	/
0.1 M KOH	Coconut Shells (Borghei et al., 2017)	1,000°C	1260 m^2^ g^−1^	−0.02/0.05 V (vs. Ag/AgCl)
0.1 M KOH	NiIn_2_S_4_/CNFs (Fu et al., 2019)	/	196.3 m^2^ g^−1^	1.46/1.50 V (vs. RHE)
0.1 M KOH	Cattail Fibers (Liu et al., 2019)	900°C	1773 m^2^ g^−1^	0.92/0.45 V (vs. RHE)
0.1 M KOH	Mulberry Leaves (He et al., 2019)	800°C	1689 m^2^ g^−1^	0.86/0.88 V (vs. RHE)
0.5 M KOH	Waste Leather (Alonso-Lemus et al., 2016)	/	2100 m^2^ g^−1^	0.905/1.050 V (vs. RHE)
0.1 M KOH	NCAC-Co (Liu et al., 2018)	800°C	1877.3 m^2^ g^−1^	0.795/0.760 V (vs. RHE)
Phosphate Buffer	Corncob (Li et al., 2018)	650°C	655.89 m^2^ g^−1^	−0.13/−0.05 V (vs. Ag/AgCl)
Phosphate Buffer	Sewage Sludge (Yuan et al., 2013)	900°C	44 m^2^ g^−1^	0.11/0.09 V (vs. RHE)
Phosphate Buffer	Egg (Lu et al., 2017)	900°C	703.47 m^2^ g^−1^	0.257/0.157 V (vs. Ag/AgCl)
Phosphate Buffer	Bacteria (Ma et al., 2017)	800°C	1926.7 m^2^ g^−1^	1.01/1.01 V (vs. RHE)
0.1 M KOH	Bagasse (Yuan et al., 2016)	1,000°C	1284 m^2^ g^−1^	1285/0.06 V (vs. Hg/HgO)
0.5 M H_2_SO_4_				0.43/0.65 V (vs. Ag/AgCl)
0.1 M KOH	Enoki Mushroom (Guo et al., 2015)	900°C	305.3 m^2^ g^−1^	0.94/0.98 V
0.5 M H_2_SO_4_				0.81/0.93 V (vs. RHE)
0.1 M KOH	Sewage Sludge (Yuan and Dai, 2016)	800°C	265.05 m^2^ g^−1^	0.05/−0.08 V
0.5 M H_2_SO_4_				0.57/0.65 V (vs. Ag/AgCl)
0.1 M KOH	Corn Starch (Wang et al., 2012)	500°C	1568.85 m^2^ g^−1^	−0.03/0.02 V
0.5 M H_2_SO_4_				0.62/0.66 V (vs. RHE)
0.1 M KOH	Starch (Yao et al., 2016)	180°C	976.6 m^2^ g^−1^	0.955/0.932 V
0.5 M H_2_SO_4_				0.840/0.930 V (vs. RHE)

## Conclusions and Outlook

In recent years, there have been great progresses to develop biomass-derived carbon ORR electrocatalysts for meeting the requirements of high performance. Some materials, especially materials derived from biomass materials, have comparable or superior ORR properties and better stability than commercial Pt/C. Thus, biomass-derived carbons have attracted particular interest as a potential substitute for commercial Pt/C due to their good activity, low-cost, and reproducibility (Gasteiger and Markovic, 2009). In addition to the biomass electrocatalysts mentioned in this minireview, there will be numerous high-performance biomass-derived electrocatalysts for practical application in the future.

Although biomass-derived carbon materials have the widest sources and the lowest price, the controllability toward distribution of active sites is very general, which depends on the composition and structure of the biomass itself. How to achieve large-scale production is also an urgent problem preventing industrialization. Up to now, most biomass-derived carbon materials are only suitable for catalyzing oxygen reduction reactions under alkaline conditions, and their performance is unsatisfying in neutral and acidic media, which seriously affects the large-scale application of fuel cells.

## Author Contributions

MW, SW, and WK wrote the manuscript. HY and ZL modified the manuscript. SY and GL supervised the manuscript.

### Conflict of Interest

The authors declare that the research was conducted in the absence of any commercial or financial relationships that could be construed as a potential conflict of interest.

## References

[B1] Alonso-Lemus I. L. Rodriguez-Varela F. J. Figueroa-Torres M. Z. Sanchez-Castro M. E. Hernandez-Ramirez A. Lardizabal-Gutierrez D. (2016). Novel self-nitrogen-doped porous carbon from waste leather as highly active metal-free electrocatalyst for the ORR. Int. J. Hydrogen Energy 41, 23409–23416. 10.1016/j.ijhydene.2016.09.033

[B2] Banham D. Ye S. Y. Knights S. Stewart S. M. Wilson M. Garzon F. (2015). UV-visible spectroscopy method for screening the chemical stability of potential antioxidants for proton exchange membrane fuel cells. J. Power Sources 281, 238–242. 10.1016/j.jpowsour.2015.02.002

[B3] Bhowmick G. D. Kibena-Poldsepp E. Matisen L. Merisalu M. Kook M. Kaarik M. (2019). Multi-walled carbon nanotube and carbide-derived carbon supported metal phthalocyanines as cathode catalysts for microbial fuel cell applications. Sustain. Energy Fuels 3, 3525–3537. 10.1039/C9SE00574A

[B4] Borghei M. Laocharoen N. Kibena-Põldsepp E. Johansson L. S. Campbell J. Kauppinen E. (2017). Porous N, P-doped carbon from coconut shells with high electrocatalytic activity for oxygen reduction: alternative to Pt-C for alkaline fuel cells. Appl. Catal. B 204, 394–402. 10.1016/j.apcatb.2016.11.029

[B5] Dai L. Xue Y. Qu L. Choi H. J. Baek J. B. (2015). Metal-Free catalysts for oxygen reduction reaction. Chem. Rev. 115, 4823–4892. 10.1021/cr5003563 25938707

[B6] Deng Q. Li X. Zuo J. Ling A. Logan B. E. (2010). Power generation using an activated carbon fiber felt cathode in an upflow microbial fuel cell. J. Power Sources 195, 1130–1135. 10.1016/j.jpowsour.2009.08.092

[B7] Fu G. Wang Y. Tang Y. Zhou K. Goodenough J. B. Lee J.-M. (2019). Superior oxygen electrocatalysis on nickel indium thiospinels for rechargeable Zn–air batteries. ACS Mater. Lett. 1, 123–131. 10.1021/acsmaterialslett.9b00093

[B8] Gasteiger H. A. Markovic N. M. (2009). Just a dream-or future reality? Science 324, 48–49. 10.1126/science.1172083 19342578

[B9] Guo C. Liao W. Li Z. Sun L. Chen C. (2015). Easy conversion of protein-rich enoki mushroom biomass to a nitrogen-doped carbon nanomaterial as a promising metal-free catalyst for oxygen reduction reaction. Nanoscale 7, 15990–15998. 10.1039/C5NR03828F 26367816

[B10] Guo S. J. Zhang S. Su D. Sun S. H. (2013). Seed-mediated synthesis of core/shell FePtM/FePt (M = Pd, Au) nanowires and their electrocatalysis for oxygen reduction reaction. J. Am. Chem. Soc. 135, 13879–13884. 10.1021/ja406091p 23978233

[B11] He D. Zhao W. Li P. Sun S. Tan Q. Han K. (2019). Bifunctional biomass-derived N, S dual-doped ladder-like porous carbon for supercapacitor and oxygen reduction reaction. J. Alloys Compd. 773, 11–20. 10.1016/j.jallcom.2018.09.141

[B12] Huang H. Wei X. Gao S. (2016). Nitrogen-doped porous carbon derived from malachium aquaticum biomass as a highly efficient electrocatalyst for oxygen reduction reaction. Electrochim. Acta 220, 427–435. 10.1016/j.electacta.2016.10.108

[B13] Jiang S. Ma Y. Jian G. Tao H. Wang X. Fan Y. (2009). Facile construction of Pt-Co/CNx nanotube electrocatalysts and their application to the oxygen reduction reaction. Adv. Mater. 21, 4953–4956. 10.1002/adma.200900677 25376105

[B14] Kaare K. Kruusenberg I. Merisalu M. Matisen L. Sammelselg V. Tammeveski K. (2016). Electrocatalysis of oxygen reduction on multi-walled carbon nanotube supported copper and manganese phthalocyanines in alkaline media. J. Solid State Electr. 20, 921–929. 10.1007/s10008-015-2990-9

[B15] Kaur P. Verma G. Sekhon S. S. (2019). Biomass derived hierarchical porous carbon materials as oxygen reduction reaction electrocatalysts in fuel cells. Prog. Mater. Sci. 102, 1–71. 10.1016/j.pmatsci.2018.12.002

[B16] Li M. Zhang H. Xiao T. Wang S. Zhang B. Chen D. (2018). Low-cost biochar derived from corncob as oxygen reduction catalyst in air cathode microbial fuel cells. Electrochim. Acta 283, 780–788. 10.1016/j.electacta.2018.07.010

[B17] Liu L. Yang X. F. Ma N. Liu H. T. Xia Y. Z. Chen C. M.. (2016). Scalable and cost-effective synthesis of highly efficient Fe2N-based oxygen reduction catalyst derived from seaweed biomass. Small 12, 1295–1301. 10.1002/smll.201503305 26753802

[B18] Liu W. Cheng S. Sun D. Huang H. Chen J. Cen K. (2015). Inhibition of microbial growth on air cathodes of single chamber microbial fuel cells by incorporating enrofloxacin into the catalyst layer. Biosens. Bioelectron. 72, 44–50. 10.1016/j.bios.2015.04.082 25957076

[B19] Liu Y. Hu M. Xu W. Wu X. Jiang J. (2019). Catalytically active carbon from cattail fibers for electrochemical reduction reaction. Front. Chem. 7:786. 10.3389/fchem.2019.00786 31799241 PMC6878766

[B20] Liu Z. Li Z. Ma J. Dong X. Ku W. Wang M. (2018). Nitrogen and cobalt-doped porous biocarbon materials derived from corn stover as efficient electrocatalysts for aluminum-air batteries. Energy 162, 453–459. 10.1016/j.energy.2018.07.175

[B21] Lu L. Yu J. Wu Z. Fan J. Lei W. Ouyang Y. (2019). Shaddock peel derived nitrogen and phosphorus dual-doped hierarchical porous carbons as high-performance catalysts for oxygen reduction reaction. Hydrogen Energy 44, 26982–26991. 10.1016/j.ijhydene.2019.08.133

[B22] Lu Y. Zhu N. Yin F. Yang T. Wu P. Dang Z.. (2017). Biomass-derived heteroatoms-doped mesoporous carbon for efficient oxygen reduction in microbial fuel cells. Biosens. Bioelectron. 98, 350–356. 10.1016/j.bios.2017.07.006 28704783

[B23] Ma X. Lei Z. Feng W. Ye Y. Feng C. (2017). Living Fe mineral@bacteria encrustation-derived and self-templated preparation of a mesoporous Fe-N-C electrocatalyst with high activity for oxygen reduction. Carbon 123, 481–491. 10.1016/j.carbon.2017.07.091

[B24] Majidi M. R. Farahani F. S. Hosseini M. Ahadzadeh I. (2019). Low-cost nanowired alpha-MnO2/C as an ORR catalyst in air-cathode microbial fuel cell. Bioelectrochemistry 125, 38–45. 10.1016/j.bioelechem.2018.09.004 30261369

[B25] Papiya F. Das S. Pattanayak P. Kundu P. P. (2019). The fabrication of silane modified graphene oxide supported Ni-Co bimetallic electrocatalysts: a catalytic system for superior oxygen reduction in microbial fuel cells. Int. J. Hydrogen Energy 44, 25874–25893. 10.1016/j.ijhydene.2019.08.020

[B26] Popat S. C. Ki D. Rittmann B. E. Torres C. I. (2012). Importance of OH(-) transport from cathodes in microbial fuel cells. ChemSusChem 5, 1071–1079. 10.1002/cssc.201100777 22615062

[B27] Qiao Y. Bao S. J. Li C. M. (2010). Electrocatalysis in microbial fuel cells-from electrode material to direct electrochemistry. Energy Environ. Sci. 3, 544–553. 10.1039/b923503e

[B28] Rajendiran R. Nallal M. Park K. H. Li O. L. Kim H.-J. Prabakar K. (2019). Mechanochemical assisted synthesis of heteroatoms inherited highly porous carbon from biomass for electrochemical capacitor and oxygen reduction reaction electrocatalysis. Electrochim. Acta 317, 1–9. 10.1016/j.electacta.2019.05.139

[B29] Sawant S. Y. Han T. H. Cho M. H. (2016). Metal-free carbon-based materials: promising electrocatalysts for oxygen reduction reaction in microbial fuel cells. Int. J. Mol. Sci. 18, 712–749. 10.3390/ijms18010025 28029116 PMC5297660

[B30] Shao M. H. Chang Q. W. Dodelet J. P. Chenitz R. (2016). Recent advances in electrocatalysts for oxygen reduction reaction. Chem. Rev. 116, 3594–3657. 10.1021/acs.chemrev.5b00462 26886420

[B31] Stamenkovic V. R. Mun B. S. Arenz M. Mayrhofer K. J. J. Lucas C. A. Wang G.. (2007). Trends in electrocatalysis on extended and nanoscale Pt-bimetallic alloy surfaces. Nat. Mater. 6, 241–247. 10.1038/nmat1840 17310139

[B32] Tang J. Wang Y. Zhao W. Zeng R. J. Liu T. Zhou S. (2019). Biomass-derived hierarchical honeycomb-like porous carbon tube catalyst for the metal-free oxygen reduction reaction. J. Electroanal. Chem. 847:113230 10.1016/j.jelechem.2019.113230

[B33] Wang D. Xin H. L. Wang H. Yu Y. Rus E. Muller D. A. (2012). Facile synthesis of carbon-supported pd-co core-shell nanoparticles as oxygen reduction electrocatalysts and their enhanced activity and stability with monolayer Pt decoration. Chem. Mater. 24, 2274–2281. 10.1021/cm203863d

[B34] Wang M. Ma J. Yang H. Lu G. Yang S. Chang Z. (2019). Nitrogen and cobalt co-coped carbon materials derived from biomass chitin as high-performance electrocatalyst for aluminum-air batteries. Catalysts 9:954 10.3390/catal9110954

[B35] Winter M. Brodd R. J. (2004). What are batteries, fuel cells, and supercapacitors? Chem. Rev. 104, 4245–4269. 10.1021/cr020730k 15669155

[B36] Wu X. Chen K. Lin Z. Zhang Y. Meng H. (2019). Nitrogen doped graphitic carbon from biomass as non noble metal catalyst for oxygen reduction reaction. Mater. Today Energy 13, 100–108. 10.1016/j.mtener.2019.05.004

[B37] Xu L. Fan H. Huang L. Xia J. Li S. Li M. (2017). Chrysanthemum-derived N and S co-doped porous carbon for efficient oxygen reduction reaction and aluminum-air battery. Electrochim. Acta 239, 1–9. 10.1016/j.electacta.2017.04.002

[B38] Yang H. Kou S. Li Z. Chang Z. Wang M. Liu Z. (2019). 3D interconnected nitrogen-self-doped carbon aerogels as efficient oxygen reduction electrocatalysts derived from biomass gelatin. RSC Adv. 9, 40301–40308. 10.1039/C9RA07926B PMC9076190 35542688

[B39] Yao W.-T. Yu L. Yao P.-F. Wei K. Han S.-L. Chen P. (2016). Bulk production of nonprecious metal catalysts from cheap starch as precursor and their excellent electrochemical activity. ACS Sustain. Chem. Eng. 4, 3235–3244. 10.1021/acssuschemeng.6b00269

[B40] Yuan S.-J. Dai X.-H. (2016). Efficient sewage sludge-derived bi-functional electrocatalyst for oxygen reduction and evolution reaction. Green Chem. 18, 4004–4011. 10.1039/C5GC02729B

[B41] Yuan W. Xie A. Li S. Huang F. Zhang P. Shen Y. (2016). High-activity oxygen reduction catalyst based on low-cost bagasse, nitrogen and large specific surface area. Energy 115, 397–403. 10.1016/j.energy.2016.09.026

[B42] Yuan Y. Yuan T. Wang D. M. Tang J. H. Zhou S. G. (2013). Sewage sludge biochar as an efficient catalyst for oxygen reduction reaction in an microbial fuel cell. Bioresour. Technol. 144, 115–120. 10.1016/j.biortech.2013.06.075 23859987

[B43] Zhao J. Liu Y. Quan X. Chen S. Yu H. Zhao H. (2017). Nitrogen-doped carbon with a high degree of graphitization derived from biomass as high-performance electrocatalyst for oxygen reduction reaction. Appl. Surf. Sci. 396, 986–993. 10.1016/j.apsusc.2016.11.073

[B44] Zheng Y. Yang D.-S. Kweun J. M. Li C. Tan K. Kong F. (2016). Rational design of common transition metal-nitrogen-carbon catalysts for oxygen reduction reaction in fuel cells. Nano Energy 30, 443–449. 10.1016/j.nanoen.2016.10.037

