# Supplementary material for: Carbon-Based Electrocatalysts Derived From Biomass for Oxygen Reduction Reaction: A Minireview
Source: Front Chem. 2020 Feb 28;8:116. doi: 10.3389/fchem.2020.00116 (PMC7059099; doi:10.3389/fchem.2020.00116)
Supplement: Supplementary file 1 [file Data_Sheet_1.DOCX]

**Electronic Supplementary Material (ESI) for**

**Carbon-based Electrocatalysts Derived from Biomass for Oxygen Reduction Reaction: A Minireview**

Mi Wang^1^, Shiyu Wang^1^, Haoqi Yang^2^, Wen Ku^2^, Shuchen Yang^1,^*, Zhenning Liu^2^, Guolong Lu^2,^*

1 Engineering College, Changchun Normal University, Changchun, Jilin Province, 130022, P. R. China.

2 Key Laboratory of Bionic Engineering (Ministry of Education), College of Biological and Agricultural Engineering, Jilin University, Changchun, Jilin Province, 130022, P. R. China.

Corresponding author:

* Guolong Lu, E-mail: Guolonglu@jlu.edu.cn.

* Shuchen Yang, E-mail: [Ysc2017@mail.cncnc.edu.cn](mailto:Ysc2017@mail.cncnc.edu.cn).

**FIGURES**

FIGURE S1 (A) Flow chart for the preparation of NHPCF from cattail spikes;

(B) Flow chart for the fabrication of NiIn_2_S_4_/CNFs;

(C) Schematic diagram for synthesis the N and Co co-doping NCAC-Co.

FIGURE S2 (A) Raman spectra of the Sewage sludge (SS) and SS-derived carbon electrocatalysts at different temperatures;

(B) a. Power density curve of MFC for various egg-derived heteroatoms-doped mesoporous carbon (EGC) electrocatalyst; b. Anode and cathode polarization curves of MFC coated with EGCs electrocatalysts.

FIGURE S3 (A) a. CV curves of two protein-rich enoki mushroom-derived carbon electrocatalysts 0.1 M KOH electrolyte; b. ORR polarization curves as-prepared electrocatalysts; c. ORR polarization curves in O_2_-saturated 0.1 M KOH electrolyte at different speeds; d. ORR polarization curves of N-C@CNT-900 at different speeds;

(B) Schematic illustration for preparation of N-C@CNT-900.


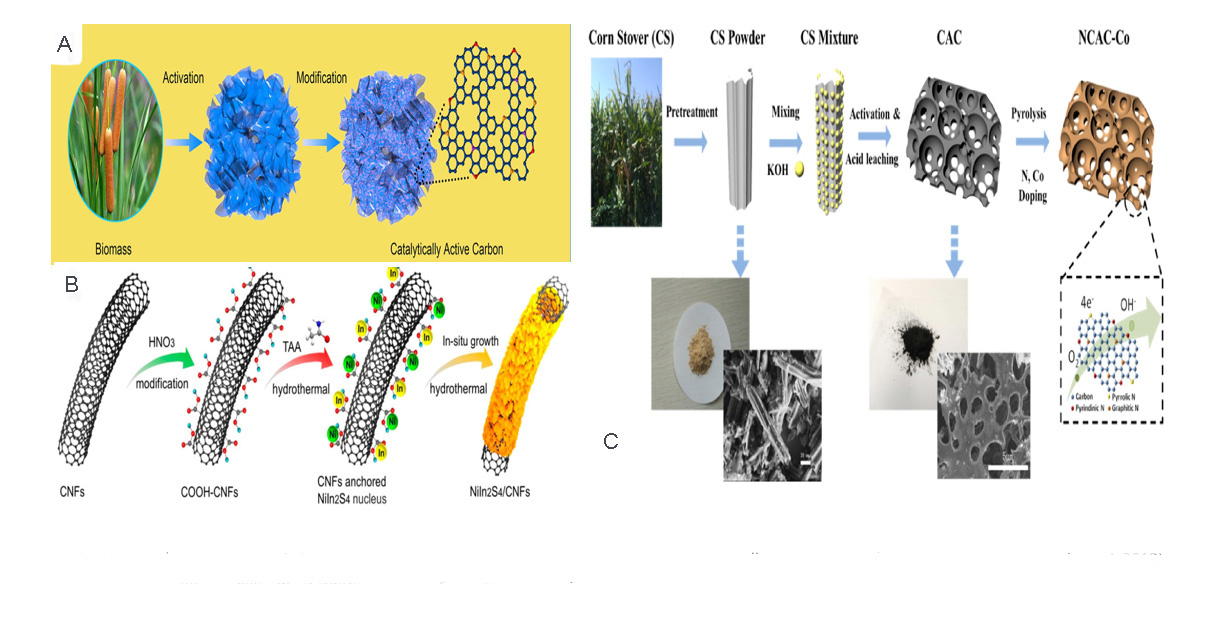


FIGURE S1 | (A) Flow chart for the preparation of NHPCF from cattail spikes; | (B) Flow chart for the fabrication of NiIn_2_S_4_/CNFs; | (C) Schematic diagram for synthesis the N and Co co-doping NCAC-Co.


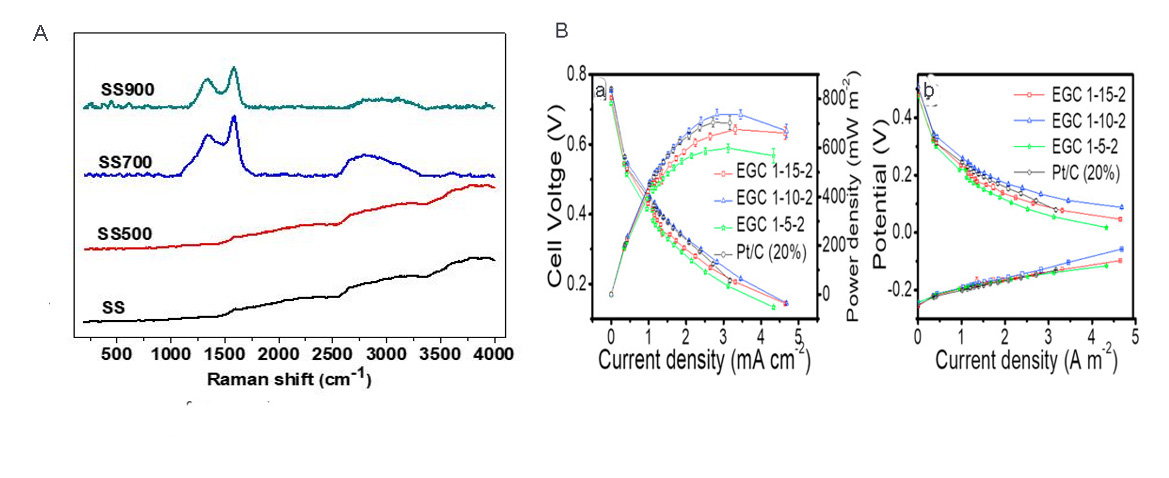


FIGURE S2 | (A) Raman spectra of the Sewage sludge (SS) and SS-derived carbon electrocatalysts at different temperatures; | (B) a. Power density curve of MFC for various egg-derived heteroatoms-doped mesoporous carbon (EGC) electrocatalyst; b. Anode and cathode polarization curves of MFC coated with EGCs electrocatalysts.


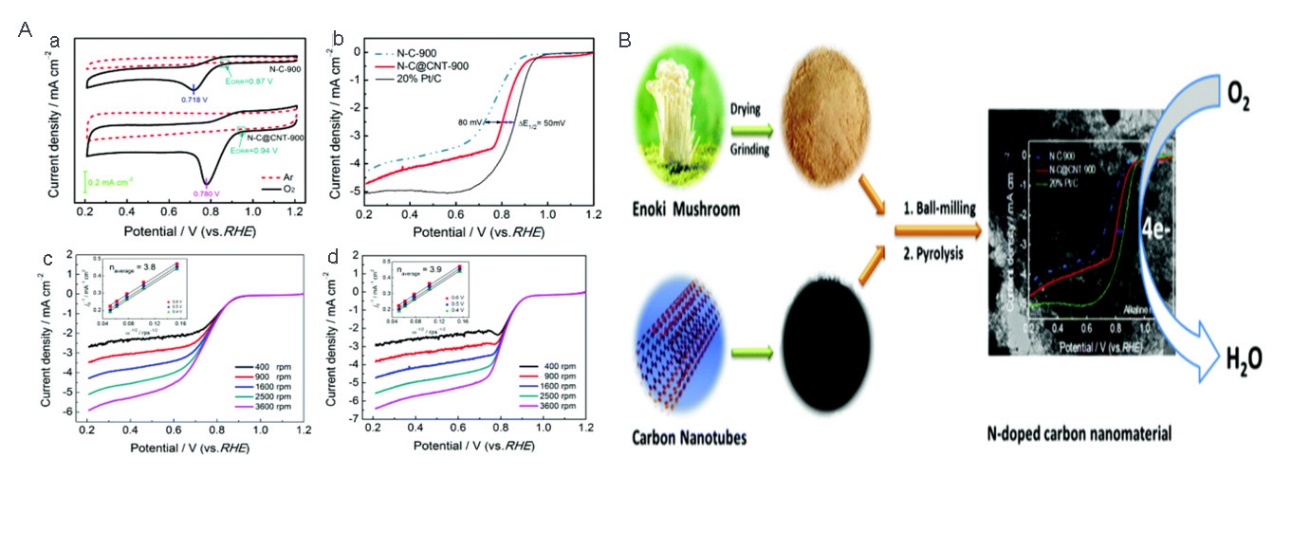


FIGURE S3 | (A) a. CV curves of two protein-rich enoki mushroom-derived carbon electrocatalysts 0.1 M KOH electrolyte; b. ORR polarization curves as-prepared electrocatalysts; c. ORR polarization curves in O_2_-saturated 0.1 M KOH electrolyte at different speeds; d. ORR polarization curves of N-C@CNT-900 at different speeds; | (B) Schematic illustration for preparation of N-C@CNT-900.
